# Effects of vibration therapy for post-stroke spasticity: a systematic review and meta-analysis of randomized controlled trials

**DOI:** 10.1186/s12938-023-01176-x

**Published:** 2023-12-12

**Authors:** Duchun Zeng, Wei Lei, Yurou Kong, Fenghao Ma, Kun Zhao, Xiangming Ye, Tongcai Tan

**Affiliations:** 1 Center for Rehabilitation Medicine, Rehabilitation & Sports Medicine Research Institute of Zhejiang Province, Department of Rehabilitation Medicine, Zhejiang Provincial People’s Hospital (Affiliated People’s Hospital, Hangzhou Medical College), No. 158, Shangtang Road, Hangzhou, 310014 China; 2 Department of Physiotherapy, Shanghai Sunshine Rehabilitation Center, Tongji University School of Medicine, Shanghai, China

**Keywords:** Post-stroke spasticity, Vibration therapy, Spasticity, Pain, Meta-analysis

## Abstract

**Background:**

The efficacy of vibration therapy (VT) in people with post-stroke spasticity (PSS) remains uncertain. This study aims to conduct a comprehensive meta-analysis to assess the effectiveness of VT in PSS.

**Methods:**

PubMed, Embase, Cochrane Library, Physiotherapy Evidence Database, and Web of Science were searched from inception to October 2022 for randomized controlled trials (RCTs) of VT in people with PSS. The primary outcome was spasticity, and secondary outcomes included pain, motor function, gait performance, and adverse events. A meta‑analysis was performed by pooling the standardized mean difference (SMD) with 95% confidence intervals (CI).

**Results:**

A total of 12 studies met the inclusion criteria. Overall, VT had significant effects on reducing spasticity (SMD = − 0.77, 95% CI − 1.17 to − 0.36, *P* < 0.01) and pain (SMD =  − 1.09, 95% CI − 1.74 to − 0.45, *P* < 0.01), and improving motor function (SMD = 0.42, 95% CI 0.21 to 0.64, *P* < 0.01) in people with PSS. However, VT had no significant effect on gait performance (SMD =  − 0.23, 95% CI − 0.56–0.10). In addition, subgroup differences in short-term anti-spasticity effects between different vibration subtypes, vibration frequencies, vibration durations, frequency of sessions, control therapy, spasticity distribution, and population classification were not significant.

**Conclusion:**

We found that VT significantly alleviated spasticity and pain in people with PSS and improved motor function, but its effect on gait performance was unclear. However, further studies are needed to validate these findings.

**Supplementary Information:**

The online version contains supplementary material available at 10.1186/s12938-023-01176-x.

## Background

Post-stroke spasticity (PSS) is a positive syndrome resulting from upper motor neuron injury, characterized by varying degrees of increased muscle tension, stretch reflex and tendon reflex hyperactivity, and is considered one of the most common functional disorders after stroke [1]. Its incidence varies between 30% and 80% depending on the statistical methods used [2]. Although lower limb spasticity may have a potentially "positive effect" (e.g., it may help the patient stand despite concomitant lower limb weakness), the same does not necessarily apply to the upper limbs. However, when PSS causes harm, effective intervention and management are required [3, 4]. The "negative effect" of PSS can lead to contracture, pain, weakness, abnormal posture and gait, activity limitation, and participation limitation, which not only directly affect functional recovery and quality of life of stroke patients but also increase the burden on family and society [5–7].

Although oral antispasmodic drugs and botulinum toxin injections have been shown to provide long-term efficacy in people with PSS, these interventions can cause an economic burden and adverse drug reactions in individuals [8]. In fact, the treatment and management of PSS should follow the principle of ladder progression [2, 9]. Therefore, the potential advantages of non-drug therapies in clinical application are gradually highlighted [10, 11]. Vibration therapy (VT) is a physical therapy that uses mechanical vibration waves to stimulate the human neuromuscular system to achieve therapeutic effect [12], showing a promising application prospect in the rehabilitation of dysfunctions after stroke. At present, VT is mainly divided into whole-body vibration (WBV) and local muscle vibration (LMV). WBV is transmitted upward to the rest of the body through the contact site, which can produce vibration stimulation to multiple muscle groups of the body at the same time [13]. LMV refers to the use of a vibrating device to directly contact and stimulate the target muscle (spasmodic muscle or antagonistic muscle) to produce a therapeutic effect [14].

Previous meta-analyses of VT for PSS have analyzed only a single vibration subtype, either WBV [15, 16] or LMV [14, 17], and have yielded mixed results. Furthermore, there is currently no consensus on the clinical practice of VT for PSS, and the choice of appropriate parameters typically depends on the therapist's best judgment. Previous meta-analyses have not comprehensively analyzed the efficacy of different VT parameters, such as vibration type, duration, sessions, spasticity location, and control treatment. Additionally, previous studies have focused solely on the efficacy of VT for spasticity in people with PSS [14–17], without considering other functional outcomes, such as pain, motor function, and gait. Therefore, our aim is to comprehensively analyze the effects of VT in people with PSS and to further explore participant characteristics, intervention characteristics, control therapy, and spasticity distribution on the anti-spasticity effect of VT.

## Results

### Search results

**Fig. 1:**
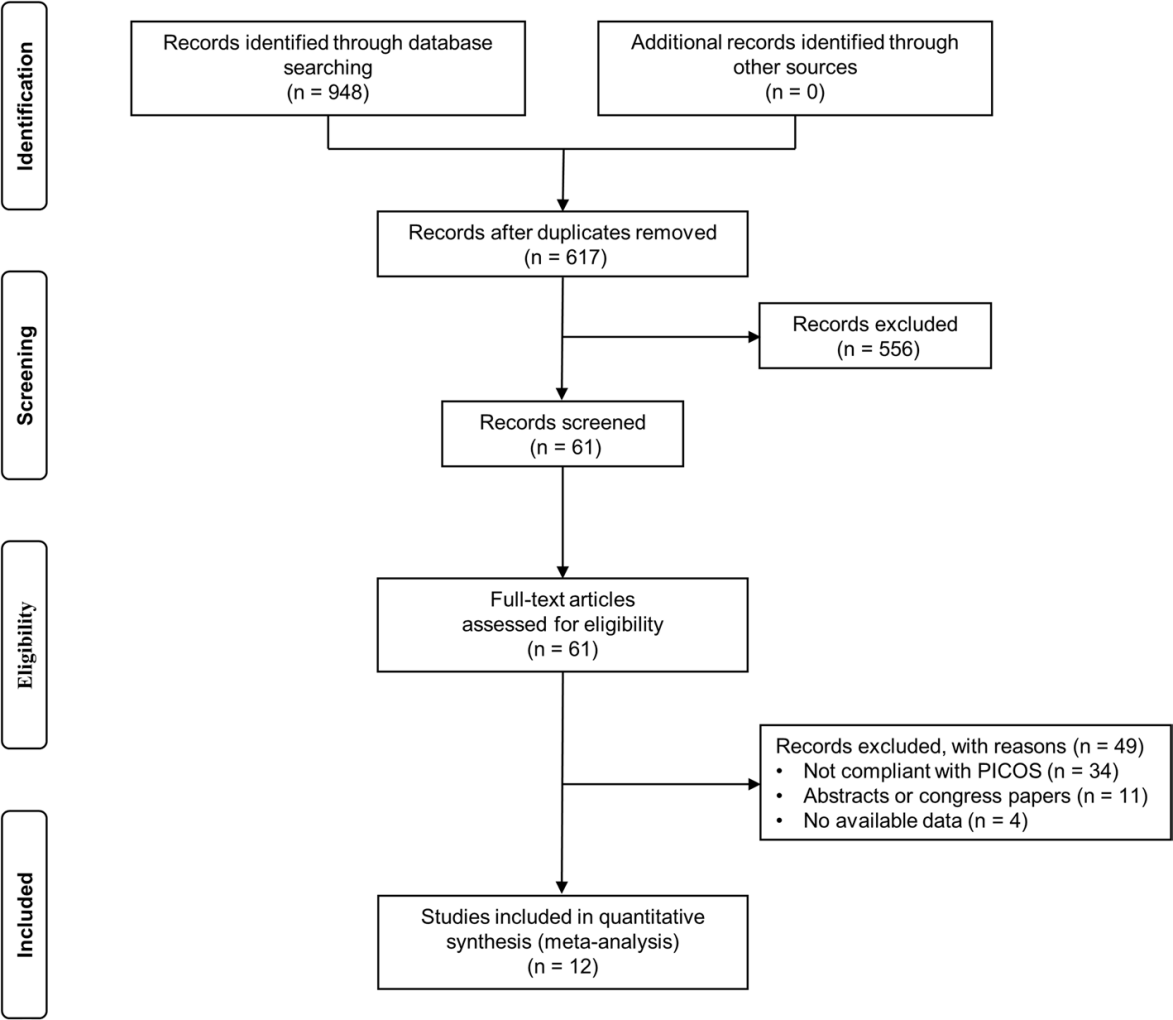
Flowchart of study selection

The study selection flowchart is presented in Fig. 1. A total of 948 articles were identified through the literature search. After removing duplicates, 617 articles remained and were further screened based on their titles and abstracts, resulting in the exclusion of 556 articles. The full text of 61 articles was assessed for eligibility, of which 12 were included in the quantitative synthesis (meta-analysis).

### Study characteristics

The characteristics of the included studies are summarized in Table 1. A total of 13 trials from 12 studies were included, with a total of 442 participants. Based on the region where the trials were conducted, the populations can be roughly divided into European [18–25] and Asian [26–29]. Four of the 12 studies did not report the specific type of stroke [18, 20, 22, 23], while one study did not report the side of lesion [22]. The time since stroke was not described in 2 studies [18, 24]. The subtypes of VT were categorized as WBV [24–29] and LMV [18–23]. Regarding the vibration frequency, 4 trials were ≤ 20 Hz and 9 trials were > 20 Hz. The duration of a single vibration was categorized as 5 min, 5–30 min, or 30 min. One trial had a single session, while 12 trials had multi-sessions. Regarding the frequency of sessions, 2 trials had 2 days/week, 8 trials had 3 days/week, and 2 trials had 5 days/week. Regarding control therapy, 7 trials were classified as sham VT, and 6 trials were classified as none VT. The distribution of spasticity included the upper limbs (shoulder, elbow, wrist) and the lower limbs (knee, ankle).

**Table 1:** Characteristics of included studies

Study	Country	Sample size	Gender (M/F)	Age (y)	Side of lesion (L/R)	Stroke type (I/H)	Time since stroke	Type of VT	Frequency	Amplitude	Duration of each session	Frequency of sessions	Adverse events	Interested outcomes	Measurement time	Follow-up
[18] Annino et al. 2019	Italy	19	14/5	67.8 ± 8.3	9/10	NA	NA	LMV	30 Hz	2 mm	5 min	3 d/wk	NA	MAS	8 wk	
		18	15/3	69.4 ± 10.4	10/8	NA	NA	None LMV								
[24] Brogårdh et al. 2012	Sweden	16	13/3	61.3 ± 8.5	7/9	14/2	37.4 ± 31.8 mon	WBV	25 Hz	3.75 mm	5–30 min	2 d/wk	Mild muscle soreness or muscle fatigue (*n* = 15)	MAS, TUGT, 6MWT	6 wk	
		15	12/3	63.9 ± 5.8	8/7	13/2	33.1 ± 29.2 mon	Sham WBV	25 Hz	0.2 mm						
[19] Caliandro et al. 2012	Italy	28	20/8	57.42 ± 12.79	14/14	18/10	100.71 ± 82.76 mon	LMV	100 Hz	0.2–0.5 mm	30 min	3 d/wk	No	MAS, VAS, WMFT	4 wk	
		21	14/7	61.85 ± 15.74	9/12	15/7	96.4 ± 66.84 mon	Sham LMV								
[20] Casale et al. 2014	Italy	15	9/6	Age range: 48–70	12/3	NA	More than 1 y	LMV	100 Hz	2 mm	30 min	5 d/wk	NA	MAS	1 wk, 2 wk	
		15	9/6	Age range: 54–70	14/1	NA	More than 1 y	Sham LMV								
[21] Celletti et al. 2017	Italy	6	4/2	43 (30–57)	2/4	2/4	2.5(2–4) y	LMV	100 Hz	0.2–0.5 mm	30 min	2 d/wk	NA	MAS, VAS, WMFT, MI	6 wk	
		6	4/2	62.5 (46–69)	4/2	4/2	5.5(2–7) mon	None LMV								
[26] Chan et al. 2012	China	15	10/5	56.07 ± 11.04	12/3	10/5	30.40 ± 25.80 mon	WBV	12 Hz	4 mm	5–30 min	1 session	NA	MAS, VAS, TUGT, 10MWT	1 session	
		15	11/4	54.93 ± 7.45	7/8	5/10	38.87 ± 38.22 mon	Sham WBV								
[22] Costantino et al. 2017	Italy	17	11/6	62.59 ± 15.39	NA	NA	37.78 ± 27.72 mon	LMV	300 Hz	2 mm	30 min	3 d/wk	No	MAS, FMA, VNRS	4 wk	
		15	10/5	60.47 ± 16.09	NA	NA	37.78 ± 27.72 mon	Sham LMV								
[27] Dang et al. 2019	China	32	20/12	57.8 ± 8.5	14/18	NA	6.6 ± 3.6 mon	WBV	5–15 Hz	1–6 mm	30 min	5 d/wk	NA	MAS, FMA, WMFT	4 wk	
		30	19/11	60.0 ± 9.0	12/18	NA	8.1 ± 3.6 mon	None WBV								
[28] Lee et al. 2016	Korea	15	5/10	59.20 ± 7.72	9/6	7/8	7.99 ± 4.23 mon	WBV	5–15 Hz	1–6 mm	30 min	3 d/wk	NA	MAS, FMA, WMFT	4 wk	
		15	9/6	60.24 ± 6.73	5/10	8/7	6.71 ± 3.85 mon	None WBV								
[29] Liao et al. 2016	China	28	20/8	60.8 ± 8.3	20/8	16/12	8.5 ± 5.2 mon	WBV (20 Hz)	20 Hz	1 mm	5–30 min	3 d/wk	Mild knee pain (*n* = 1), fatigue (*n* = 3)	MAS, TUGT, 6MWT	10 wk	
		28	18/10	62.9 ± 10.2	19/9	16/12	8.1 ± 4.2 mon	WBV (30 Hz)	30 Hz	1 mm	5–30 min	3 d/wk	Fatigue (*n* = 2)			
		28	24/4	59.8 ± 9.1	12/16	17/11	9.0 ± 4.6 mon	Sham WBV								
[23] Marconi et al. 2011	Italy	15	9/6	63.6 ± 7.6	9/6	NA	39.9 ± 28.8 mon	LMV	100 Hz	0.2–0.5 mm	30 min	3 d	NA	MAS, MI, WMFT	3 d	1 wk, 2 wk
		15	8/7	66.3 ± 11	8/7	NA	40.6 ± 25.1 mon	None LMV								
[25] Tankisheva et al. 2014	Belgium	7	4/3	57.4 ± 13	4/3	6/1	7.71 ± 8.6 y	WBV	35 Hz & 40 Hz	1.7 mm & 2.5 mm	5–30 min	3 d/wk	No	MAS	6 wk	6 wk
		8	4/4	65.3 ± 3.7	4/4	5/3	5.28 ± 3.6 y	None WBV								

*6MWT* 6-minute walk test, *10MWT* 10-meter walk test, *FMA* Fugl-Meyer assessment, *I/H* Infarction/Hemorrhage, *L/R* Left/Right, *M/F* Male/Female, *MI* Motricity Index, *NA* not available, *TUGT* timed up and go test, *VAS* visual analog scale, *VNRS* verbal numerical rating scale, *WMFT* Wolf motor function test

### Risk of bias

**Fig. 2:**
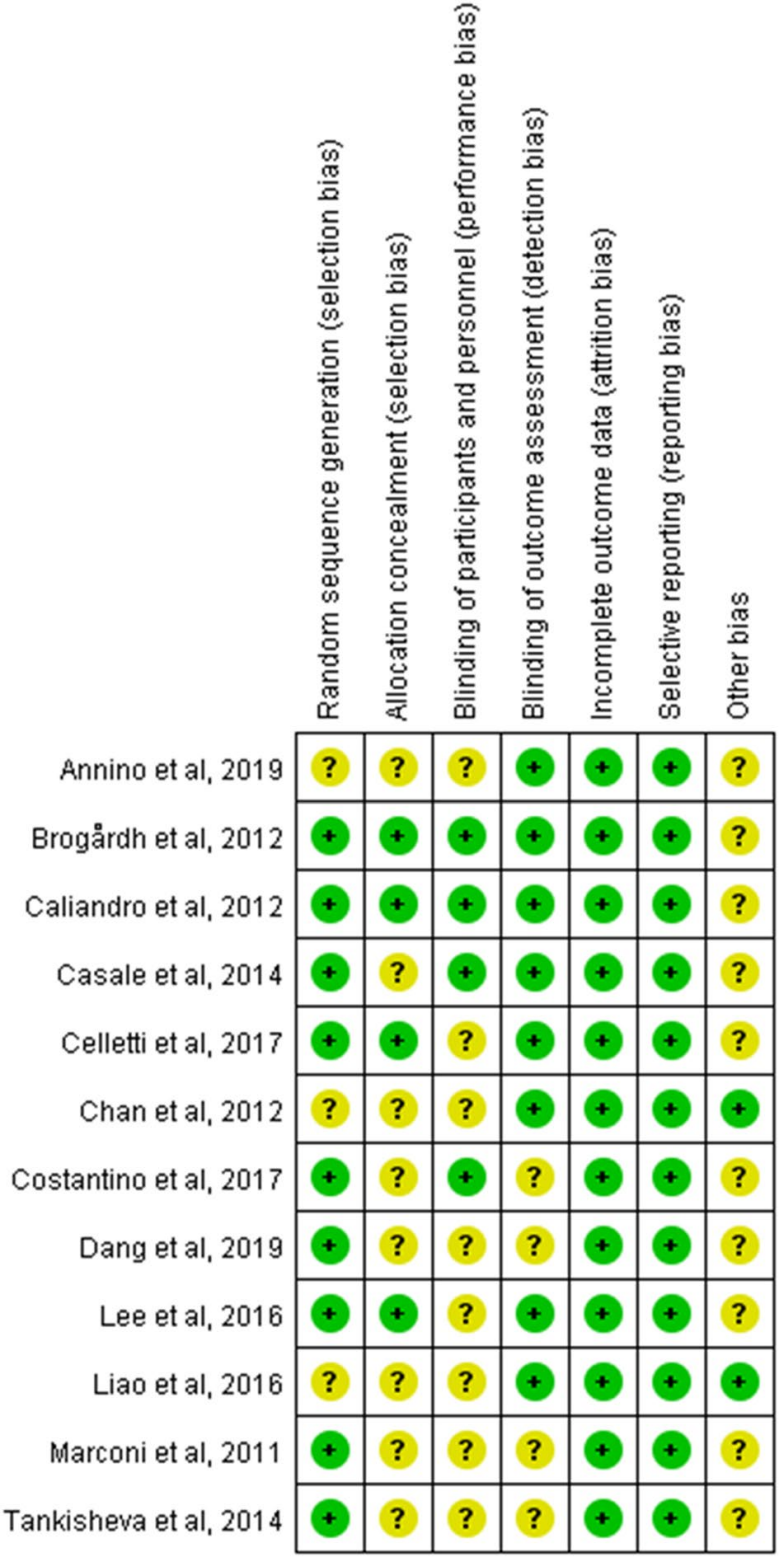
Risk of bias summary. Green: low risk of bias; Yellow: unclear risk of bias

The risk of bias is shown in Fig. 2. Nine studies had a low risk for random sequence generation, while four studies had a low risk for both allocation concealment and blinding of participants. Eight articles showed a low risk for blinding of outcome assessment, while all articles had a low risk for incomplete outcome data and selective reporting.

### Meta-analysis

#### Short-term effects of VT on spasticity

**Fig. 3:**
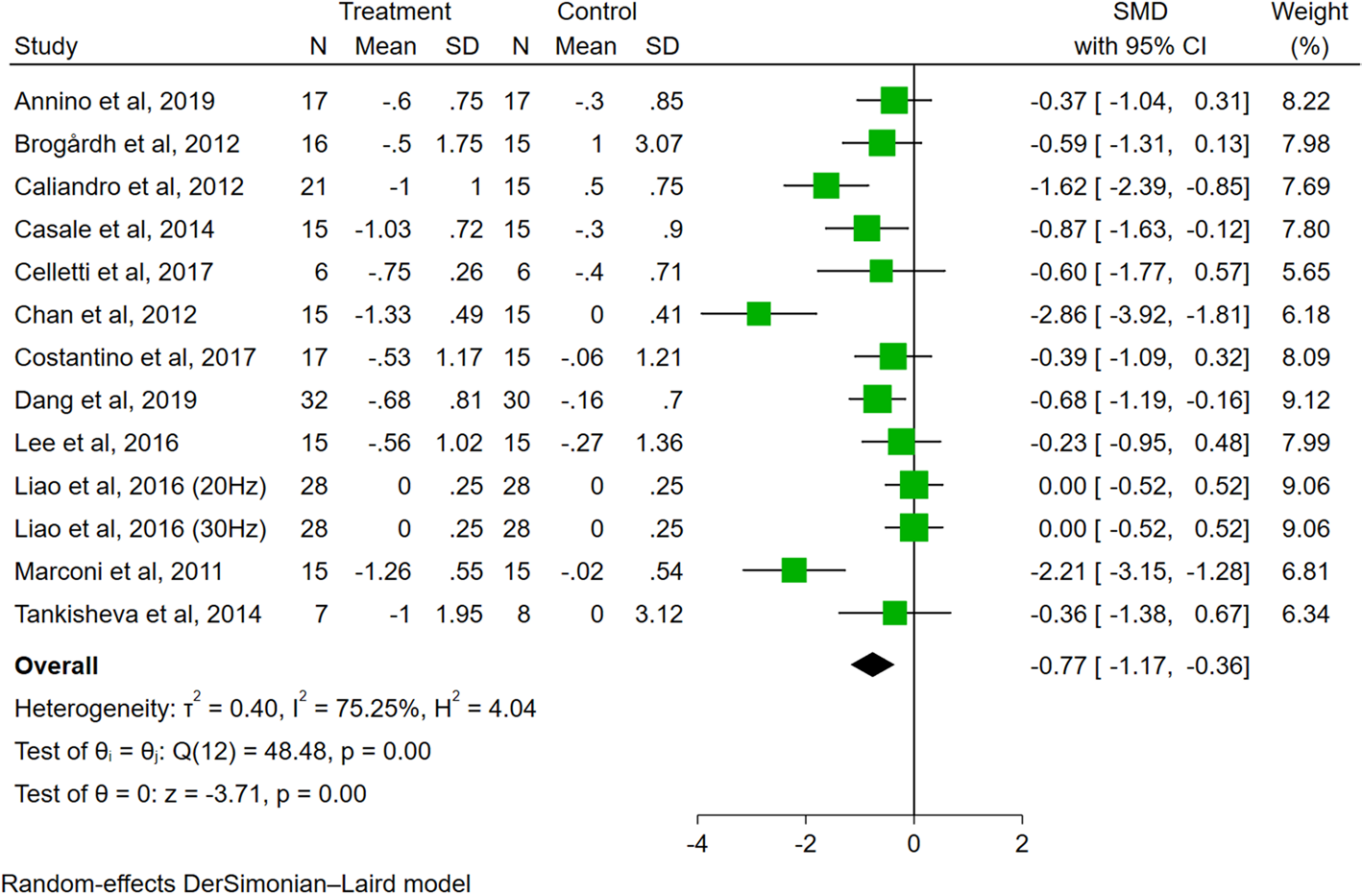
Forest plots illustrating the short-term effects of VT on spasticity

A total of 13 trials evaluated the effect of VT on spasticity. Meta-analysis showed that compared to the control group, VT had a significant effect on reducing spasticity in people with PSS (SMD = − 0.77, 95% CI − 1.17 to − 0.36, *P* < 0.01, *I*^2^ = 75.25%) (Fig. 3).

#### Subgroup of analysis

**Fig. 4:**
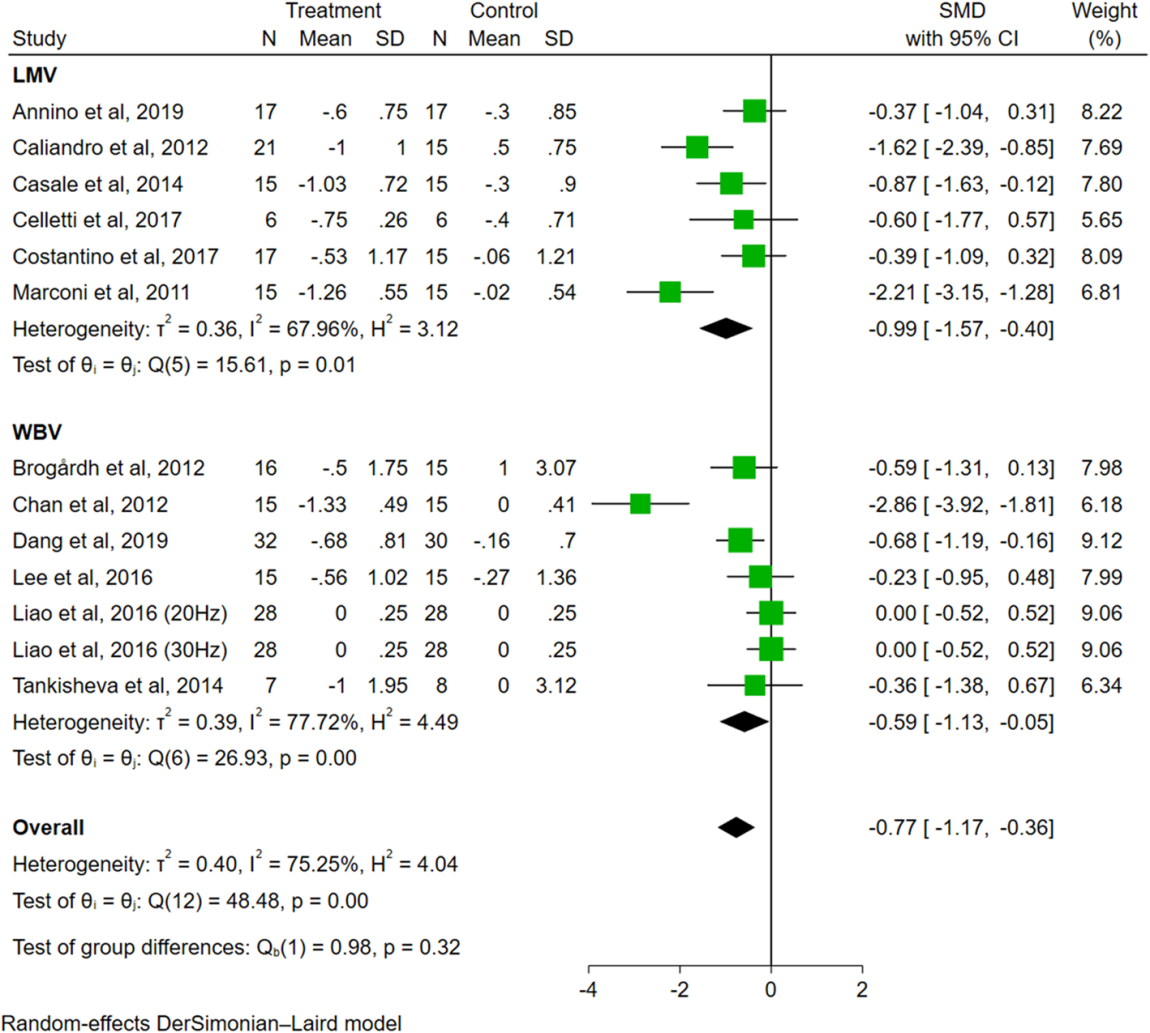
The subgroup analysis of vibration subtype (LMV vs. WBV)

In the analysis of subtypes of VT (Fig. 4), the effect sizes were significant for both the LMV group (SMD = − 0.99, 95% CI − 1.57 to − 0.40, *P* < 0.01) and WBV group (SMD = − 0.59, 95% CI − 1.13 to − 0.05, *P* = 0.03). The difference among groups was not significant (*P* = 0.32).

**Fig. 5:**
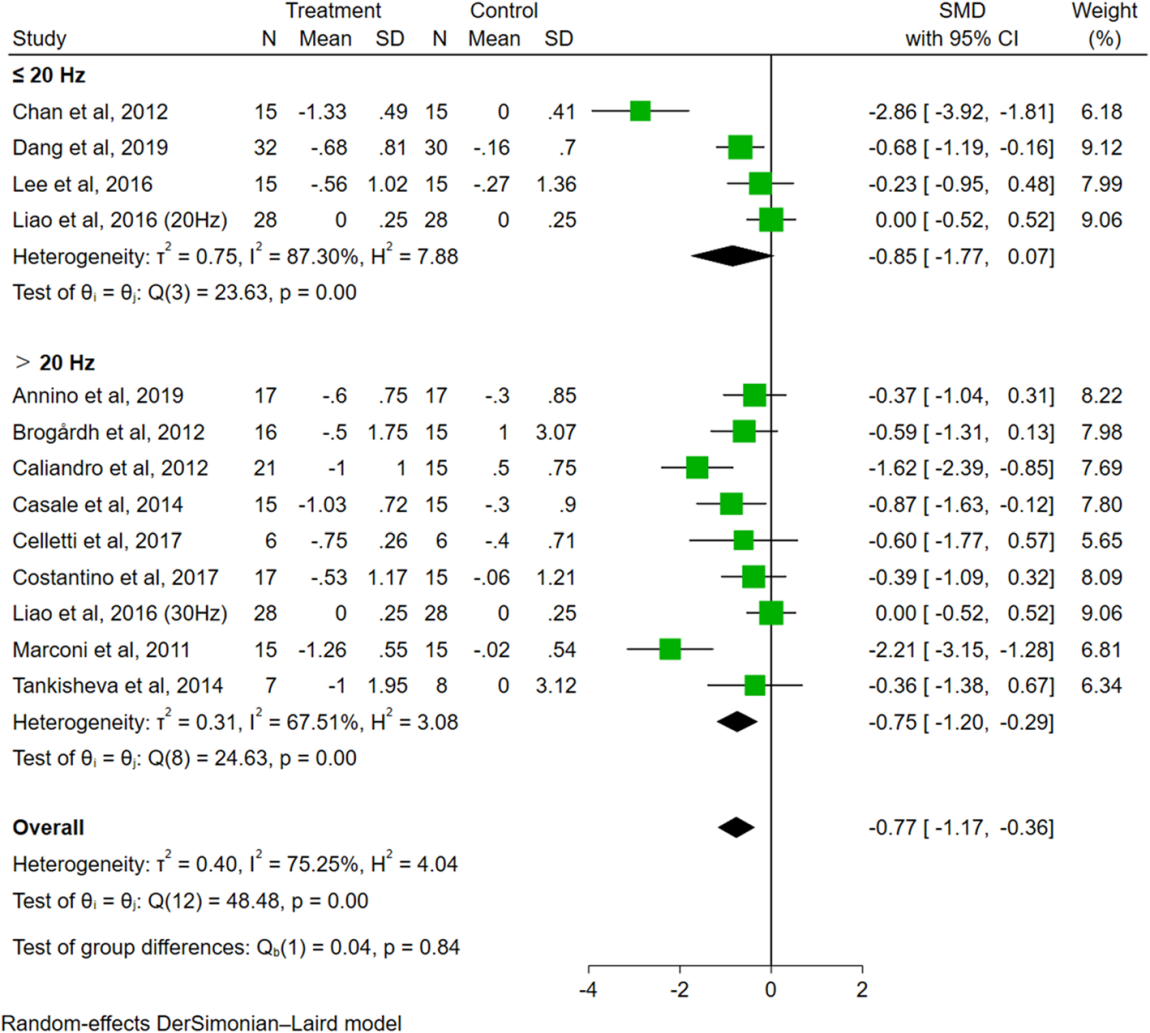
The subgroup analysis of vibration frequency (≤ 20 Hz vs. > 20 Hz)

Regarding the analysis of vibration frequency (Fig. 5), the effect size of the group with a frequency of ≤ 20 Hz (SMD = − 0.85, 95% CI − 1.77 to 0.07, *P* = 0.07) was not significant, while the effect size of the group with a frequency of > 20 Hz was significant (SMD = − 0.75, 95% CI − 1.20 to − 0.29, *P* < 0.01). The difference among frequency groups was not significant (*P* = 0.84).

**Fig. 6:**
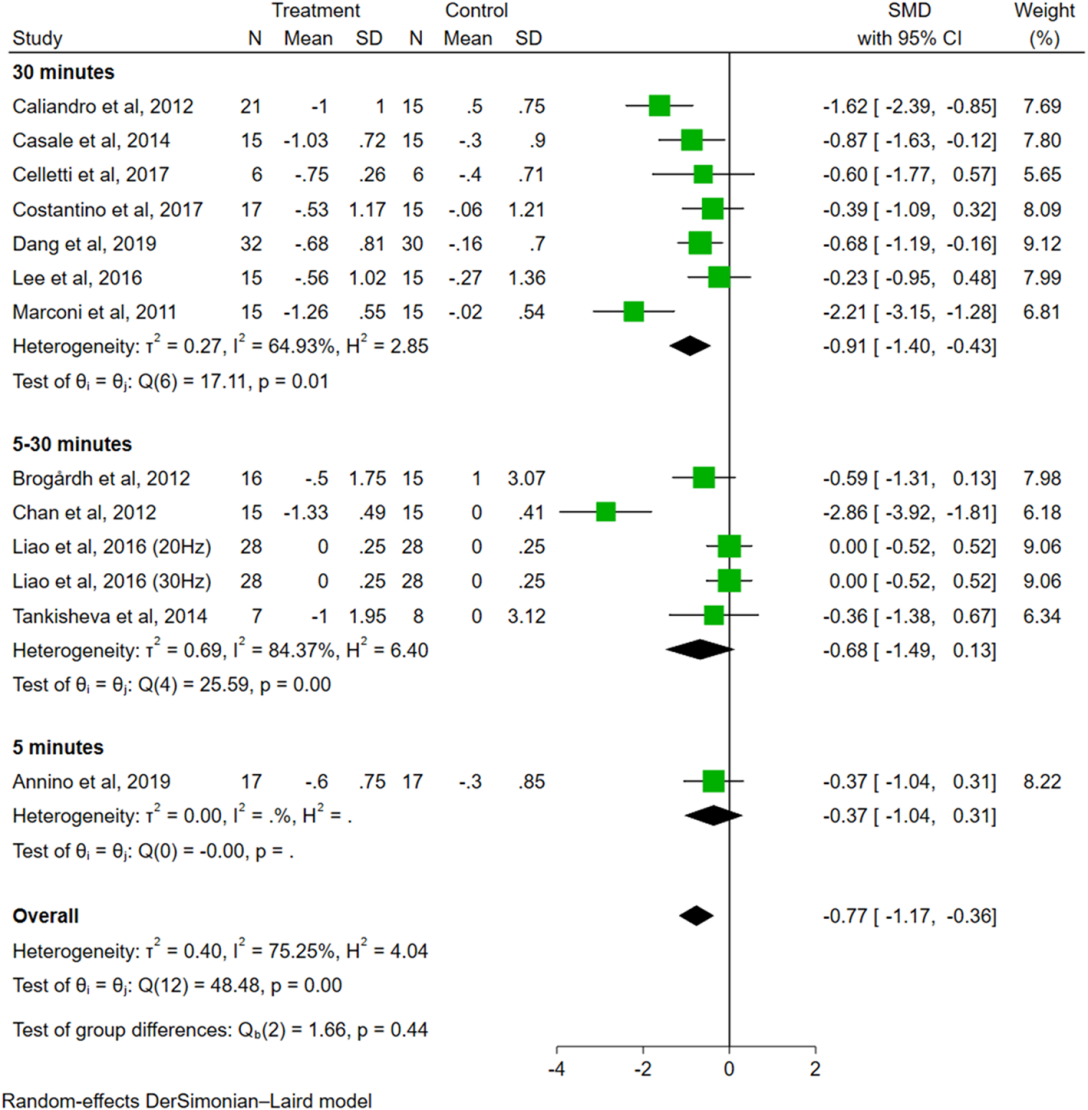
The subgroup analysis of duration of each session (30 min vs. 5–30 min vs. 5 min)

In the analysis of duration of each session (Fig. 6), the effect size of the 30 min group was significant (SMD = − 0.91, 95% CI − 1.40 to − 0.43, *P* < 0.01), while the effect sizes of the 5-30 min group (SMD = − 0.68, 95% CI − 1.49 to 0.13, *P* = 0.10) and the 5 min group (SMD = − 0.37, 95% CI − 1.04 to 0.31, *P* = 0.29) were not significant. The difference among groups was not significant (*P* = 0.44).

In the analysis of the number of sessions, a total of 12 trials evaluated the effect of multi-session of VT on spasticity, but only one trial evaluated the effect of a single session of VT on spasticity. Therefore, we performed a combined analysis for multi-session of VT only. The results showed that the effect sizes of the multi-session group was significant (SMD = − 0.61, 95% CI − 0.95 to − 0.27, *P* < 0.01, Additional file 1: Fig. S1).

**Fig. 7:**
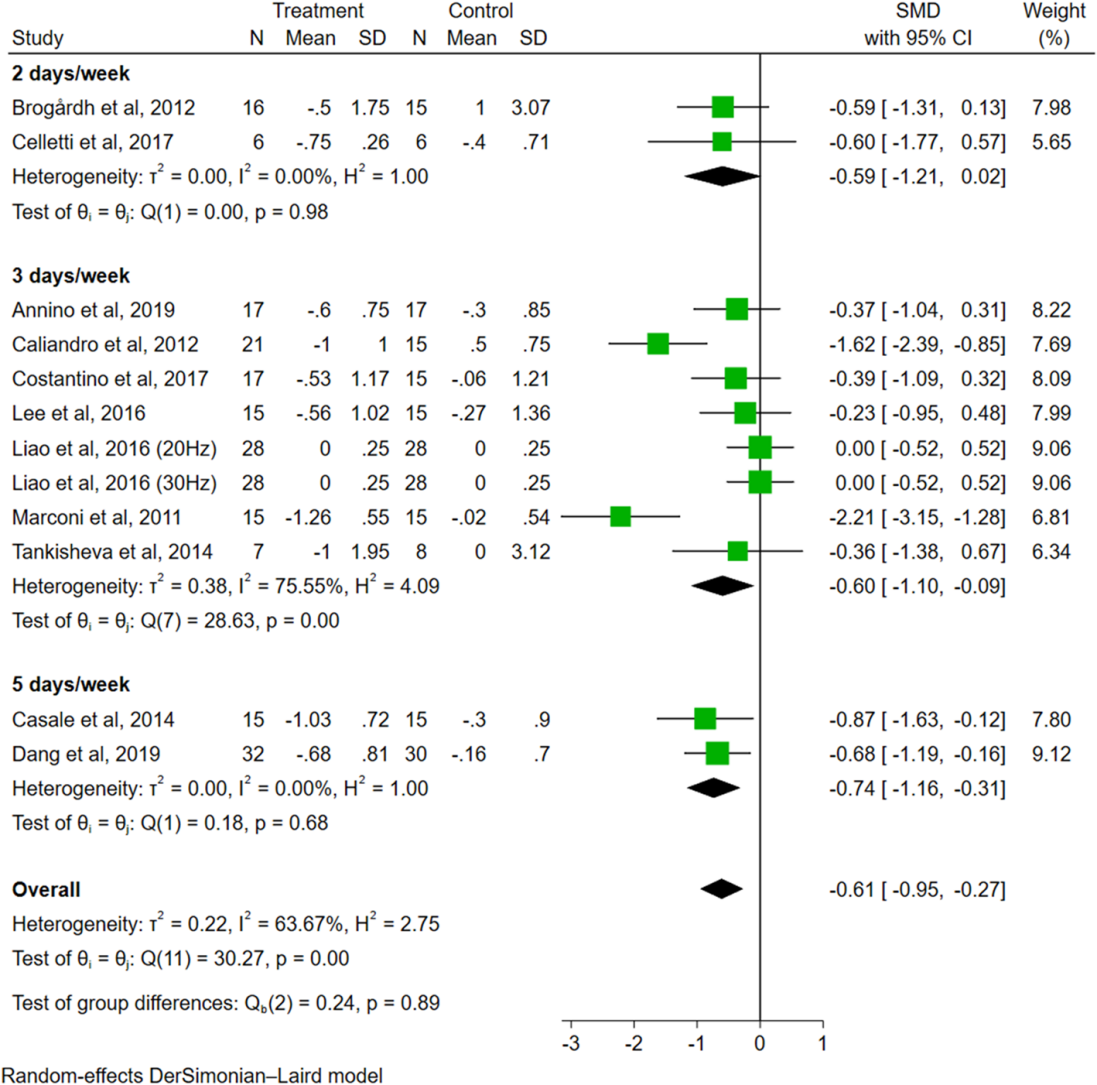
The subgroup analysis of frequency of sessions (2 days/week vs. 3 days/week vs. 5 days/week)

Regarding the analysis of frequency of sessions (Fig. 7), the effect size of the group with sessions 2 days/week (SMD = − 0.59, 95% CI − 1.21 to 0.02, *P* = 0.06) was not significant. The effect size of the groups with sessions 3 days/week (SMD = − 0.60, 95% CI − 1.10 to − 0.09, *P* = 0.02) and 5 days/week (SMD = − 0.74, 95% CI − 1.16 to − 0.31, *P* < 0.01) were significant. The difference among groups was not significant (*P* = 0.89).

**Fig. 8:**
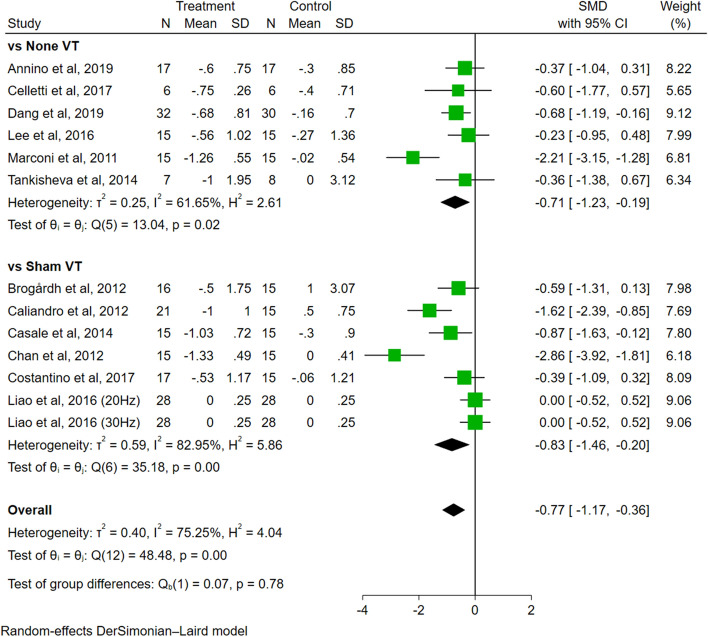
The subgroup analysis of control therapy (none VT vs. sham VT)

In the analysis of control therapy (Fig. 8), the effect sizes were not significantly different between sham VT (SMD = − 0.83, 95% CI − 1.46 to − 0.20, *P* = 0.01) and none VT (SMD = − 0.71, 95% CI − 1.23 to − 0.19, *P* = 0.01). The difference among groups was not significant (*P* = 0.78).

**Fig. 9:**
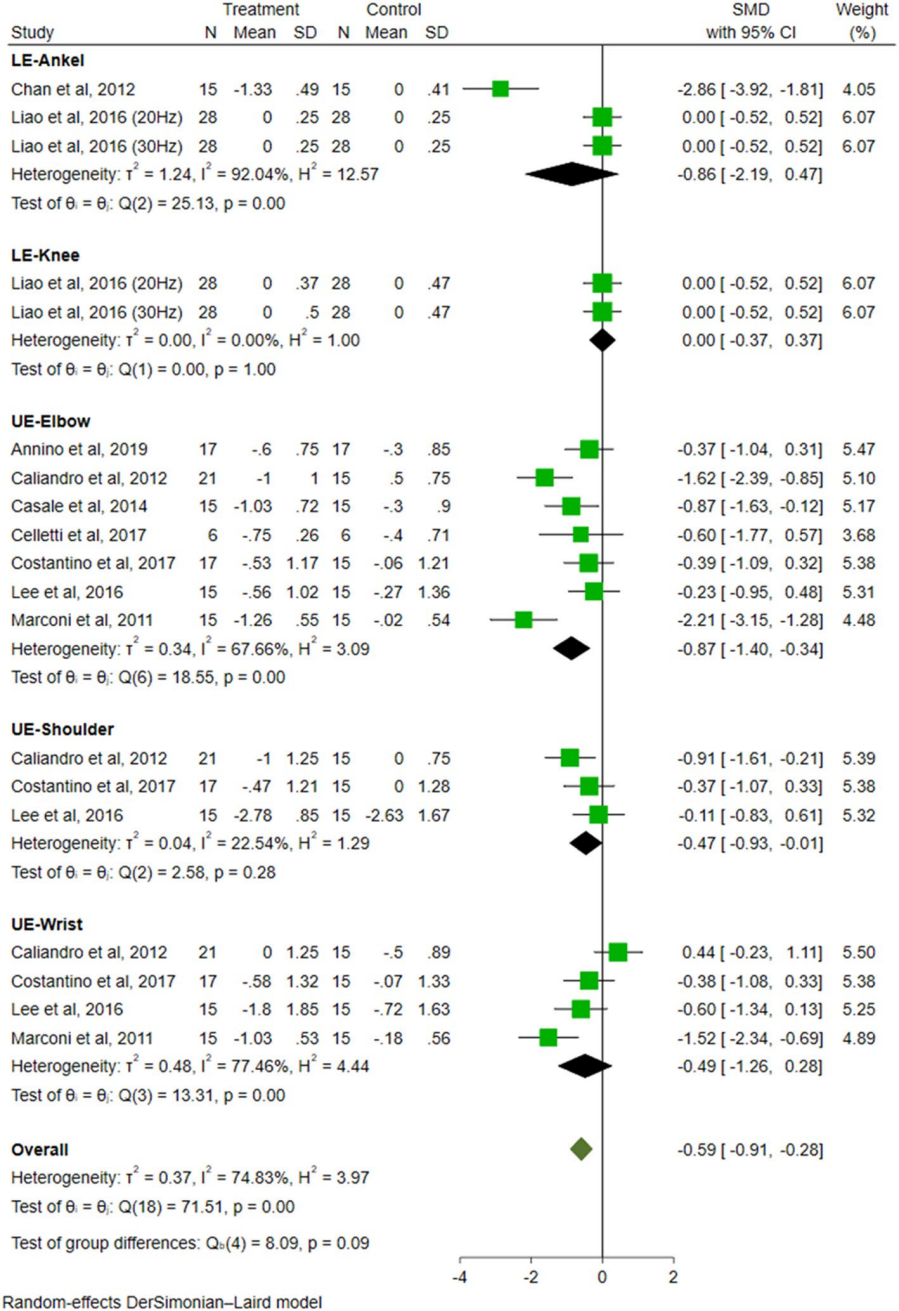
The subgroup analysis of distribution of spasticity (ankle vs. knee vs. elbow vs. shoulder vs. wrist)

Regarding the analysis of spasticity distribution (Fig. 9), the effect size was significant for the elbow (SMD = − 0.87, 95% CI − 1.40 to − 0.34, *P* < 0.01) and shoulder (SMD = − 0.47, 95% CI − 0.93 to − 0.01, *P* = 0.05), respectively. There were no statistically significant improvements in other limb parts, including wrist (SMD = − 0.49, 95% CI − 1.26 to 0.28, *P* = 0.21), ankle (SMD = − 0.86, 95% CI − 2.19 to 0.47, *P* = 0.21), and knee (SMD =  0.00, 95% CI − 0.37 to 0.37, *P* = 1.00). The difference among groups was not significant (*P* = 0.09).

**Fig. 10:**
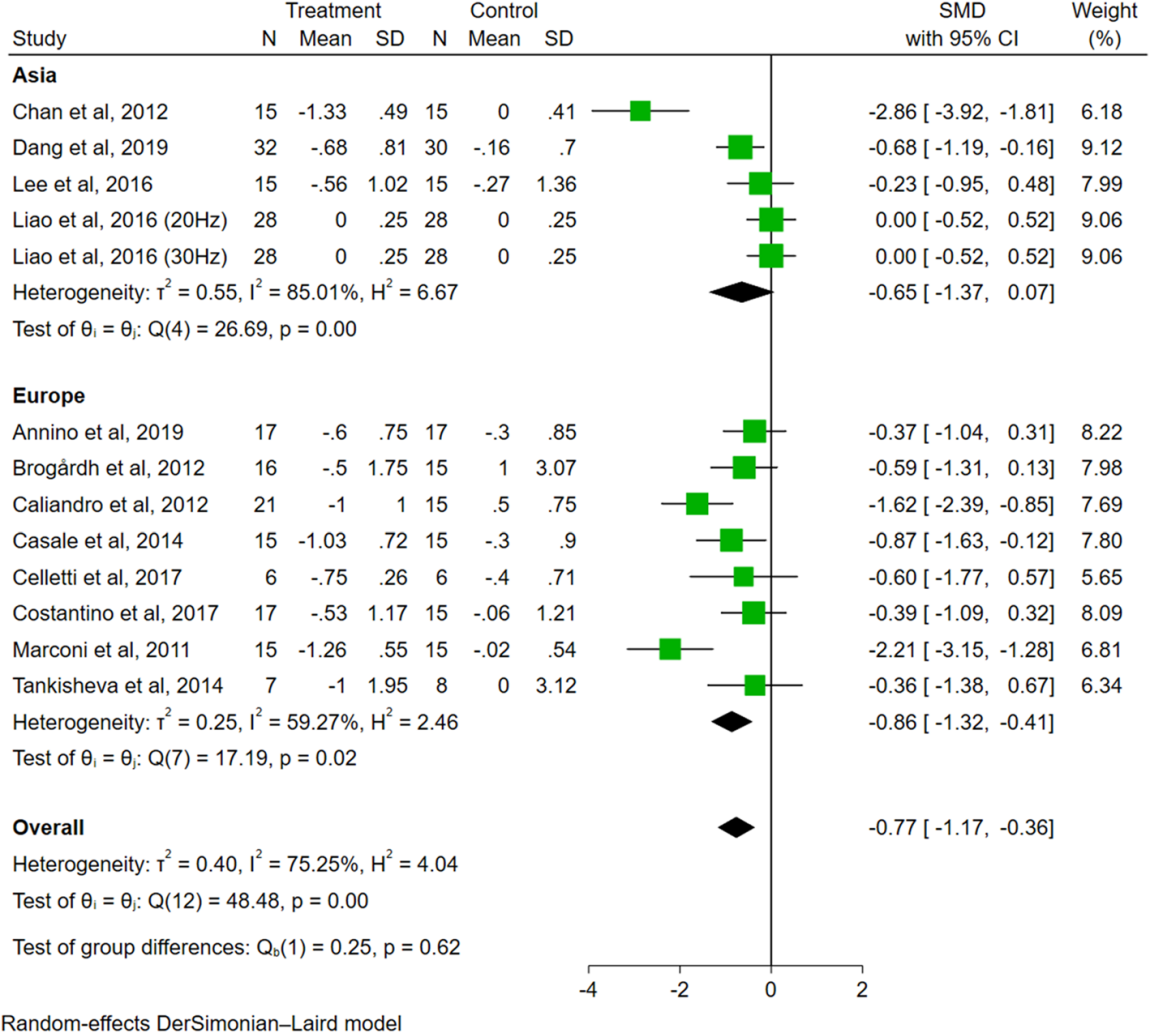
The subgroup analysis of population classification (Asian vs. European)

In the analysis of population classification (Fig. 10), the effect size of the European group was significant (SMD = − 0.86, 95% CI − 1.32 to − 0.41, *P* < 0.01), while the effect size of the Asian group was insignificant (SMD = − 0.65, 95% CI − 1.37 to 0.07, *P* = 0.08). The difference among groups was not significant (*P* = 0.62).

#### Long-term effects of VT on spasticity

For the time of assessment analysis, the effect size of the long-term group (SMD = − 0.92, 95% CI − 2.32 to 0.49, *P* = 0.20) was not found to be significant, while the effect size of the short-term group (SMD = − 0.77, 95% CI − 1.17 to − 0.36, *P* < 0.01) was found to be significant. The difference between the two groups was not significant (*P* = 0.84) (Additional file 2: Fig. S2).

#### Effects of VT on pain

Four studies evaluated the effect of VT on pain, and the results showed that VT had a significant effect on reducing pain in people with PSS compared to the control group (SMD = − 1.09, 95% CI − 1.74 to − 0.45, *P* < 0.01) (Additional file 3: Fig. S3).

#### Effects of VT on motor function

Six studies evaluated the effect of VT on motor function, and the results showed that VT had a significant effect on improving motor function in people with PSS compared to the control group (SMD = 0.42, 95% CI 0.21 to 0.64, *P* < 0.01) (Additional file 4: Fig. S4).

#### Effects of VT on gait

Four studies evaluated the effect of VT on gait, and the meta-analysis showed that VT had no significant effect on improving gait in people with PSS compared to the control group (SMD = − 0.23, 95% CI − 0.56 to 0.10) (Additional file 5: Fig. S5).

#### Adverse events

Out of the 12 studies, two reported adverse events, 3 reported no adverse events, and the remaining 7 did not provide information about adverse events. Specifically, Brogårdh et al. [24] reported that 15 participants who underwent WBV experienced temporary mild muscle soreness or muscle fatigue, but the study did not specify whether these participants were in the experimental group or the sham stimulation group. Liao et al. [29] reported that one participant in the low-frequency (20 Hz) WBV group experienced mild knee pain, and three participants reported fatigue. In the high-frequency (30 Hz) WBV group, 2 participants reported fatigue and withdrew from the study. No studies on LMV reported adverse events. As the authors did not provide specific explanations for the occurrence of adverse events, it was not possible to determine the relationship between the occurrence of these events and VT.

#### Publication bias and sensitivity analysis

The assessment of funnel plot symmetry (Additional file 6: Fig. S6) and the results of the Egger test (*P* < 0.1) both detected significant publication bias among the 12 studies included in the analysis of spasticity. Thus, we concluded that publication bias might have influenced the short-term effect of VT on spasticity. However, the sensitivity analysis showed no significant change in the results of spasticity (Additional file 7: Fig. S7), indicating that the findings were robust and reliable.

## Discussion

Based on this meta-analysis, several relevant conclusions can be drawn. Firstly, our findings demonstrate a positive short-term effect of VT on spasticity and pain reduction, as well as improvements in motor function among people with PSS. However, the impact of VT on gait performance remains uncertain. Secondly, subgroup analysis reveals that both WBV and LMV had significant anti-spasticity effects, with no significant differences observed among various vibration frequencies, durations, session frequencies, and control therapies. Thirdly, the anti-spasticity effect of VT is primarily observed in the upper limbs, particularly the shoulder and elbow regions, while its effect on the lower limbs is limited based on the distribution of spasticity. In terms of population categorization, although VT demonstrates anti-spasticity effects in European populations, the difference in effects between European and Asian populations is not statistically significant. Finally, the existing information on adverse events does not allow for a quantitative analysis of the safety of VT in people with PSS.

For spasticity, our analysis showed that VT showed a certain anti-spasticity effect. This may be due to the following potential mechanisms outlined below. First, vibration can increase the excitability of the motor cortex and produce intracortical inhibition, thereby reducing muscle tension. Marconi et al. [23] found that the activation area of M1 was increased, and the maximum motor evoked potential and short-interval intracortical inhibition were significantly improved after vibration. Lapole et al. [30] also found that the amplitude of motor-evoked potential increased significantly after vibration stimulation of the Achilles tendon. Second, vibration can cause sustained and repetitive changes in the length of muscle spindles. Pope et al. [31] observed that VT can prolong the latency of the H reflex in lower limbs and inhibit the reflex of the muscle spindle. Additionally, with the occurrence of PSS, muscles may produce a series of secondary structural changes, including collagen and elastic tissue fibrosis, shortening of muscle fiber length, and reduction of muscle thickness [32–34]. Vibration can promote the storage and release of muscle elasticity, and help to release adherent muscle tissue. Marín et al. [35] found that VT was effective in increasing the thickness of rectus femoris and vastus lateralis.

Regarding the type of vibration, our results showed that WBV and LMV had the similar effects on short-term spasticity relief. Consistent with previous meta-analyses, WBV and LMV were shown to significantly relieve spasticity, respectively [14, 16]. Interestingly, our results showed that the overall effect size of LMV tended to be slightly larger than that of WBV. We hypothesize that this may be due to the fact that LMV is often used in practice to directly target local muscles (spasmodic or antagonistic muscles), while WBV delivers vibratory stimulation through contact sites such as the foot, hand, or hip without directly stimulating the target muscles. Additionally, patients are usually in a passive state of relaxation when receiving LMV, making it difficult to perform active training simultaneously. In contrast, patients can perform some active training while receiving WBV. However, WBV may require the patient to have some postural control or balance (the ability to maintain a standing, sitting, or kneeling position on a vibrating platform), while LMV requires little postural control. Therefore, an individualized vibration type should be chosen based on treatment goals, the severity of spasticity, and the functional status of each stroke patient.

Regarding vibration frequency, the resonance frequency of some vital organs in the human body is 5–20 Hz, and vibration stimulation in this frequency range may cause damage to the human body [36, 37]. For safety reasons, we choose 20 Hz as the reference standard for frequency grouping. Our results showed that VT (WBV) ≤ 20 Hz did not significantly relieve spasticity, whereas > 20 Hz of VT did. This was inconsistent with the results of a previous study [16], which showed that WBV < 20 Hz could significantly alleviate spasticity. The possible reason for this contradictory conclusion is the inconsistency of our research methods, such as the classification criteria of vibration frequency, the inclusion of literature and participants, and the analysis models. However, our subgroup analysis also showed that there was no statistically significant difference between subgroups. This indicated that vibration frequency might not be a significant factor affecting efficacy. Liao et al. [29] conducted a three-arm study and found that neither low frequency (20 Hz) nor high frequency (30 Hz) of WBV significantly reduced spasticity. Similarly, Wei et al. [38] found that high frequency (26 Hz) of WBV had no greater benefit in balance and physical performance in patients with chronic stroke compared to low frequency (13 Hz). Therefore, future studies should further explore the role of VT with different frequencies in the efficacy of PSS treatment.

Regarding the duration of each session, our subgroup analysis showed that 30 min of VT significantly reduced spasticity, whereas 5–30 min and 5 min did not. Interestingly, the results also showed a trend of gradual increase in effect size with increasing vibration duration. We assumed that 30 min of VT could serve as an effective stimulation parameter. However, a previous meta-analysis showed that 10 min of WBV was more effective than 5 min and 15 min of WBV in patients with spasticity [16]. This difference in results may be due to different inclusion and exclusion criteria. For example, most of the articles in the previous meta-analysis were published in Chinese [16], while our study only included studies published in English. In addition, due to the limited number of relevant studies, we did not further analyze which single vibration duration is better for different vibration subtypes. Therefore, further investigation is necessary to address this inconsistency.

While recognizing the significance of subgroup analysis based on session numbers, we acknowledge the limited inclusion of only one study using a single-session approach compared to 12 studies employing multi-session interventions. This poses challenges in ensuring the comprehensive and reliable results of this analysis. Future research should aim to include a larger sample size in the single-session group to provide a more comprehensive evaluation of session numbers' impact on intervention effectiveness. Our results demonstrated the positive short-term effect of multi-session VT on relieving spasticity, potentially resulting from the cumulative impact of multiple stimulations. Although a few studies were not included in our analysis due to eligibility criteria, they offer valuable insights into single-session interventions. For instance, Noma et al. [39] observed significant decreases in the amplitude of the F wave, F/M ratio, and MAS scores for elbow and wrist flexors in stroke patients who received a single-session of LMV. Conversely, Alp et al. [40] found no significant reduction in ankle spasticity with a single-session of WBV. This discrepancy may be attributed to the differing durations of the interventions (20 min in the Noma et al. [39] study versus 5 min in the Alp et al. [40] study). Future studies should explore the potential benefits of a single session of VT. Regarding the frequency of sessions, our analysis showed that although the effect size of 3 days/week and 5 days/week was slightly larger than that of 2 days/week, the difference between groups was not significant. Additionally, due to the heterogeneity of the studies, the intervals between sessions remain unknown. Thus, current evidence indicates that the frequency of sessions may not be a significant indicator of VT efficacy.

For distribution of spasticity, our results indicated that VT significantly reduced spasticity in the upper limbs, especially in the shoulder and elbow. Previous meta-analysis showed that WBV significantly reduced upper limb spasticity [16]. Besides, another meta-analysis also found that LMV significantly reduced spasticity in the upper limbs [14]. Additionally, spasticity has been shown to inhibit active upper limb function [41], our results indicated that VT significantly improved upper limb motor function in people with PSS. We suspect that this is mainly because with the relief of spasmodic (antagonistic) muscle resistance, this provides the basis for prime muscle activation and functional training of the upper limbs. Functional recovery of the hemiplegic upper limb in stroke depends on multiple factors, including the severity of paresis, the degree of spasticity, and the degree of motor and sensory loss [42]. Therefore, we hypothesized that the potential benefits of VT in reducing spasticity and improving motor function may contribute to its wider use in upper limb function rehabilitation.

Lower limb spasticity has been shown to affect the recovery of motor and gait function in people with stroke [11]. Although there is no difference in the incidence of spasticity between the upper and lower limbs, spasticity is more severe in the upper limbs than in the lower limbs [43]. Currently, the application of LMV in people with stroke is mainly concentrated in the upper limbs, and less in the lower limbs. Lee et al. [44] observed the effect of LMV on the tibialis anterior muscle and Achilles tendon in stroke patients, and the results showed an improvement in hemiplegic gait caused by spasticity after vibration. However, the outcome measures of the study did not report an improvement in spasticity. Our results showed that WBV did not significantly reduce lower limbs spasticity (knee and ankle) and improve gait performance in people with PSS. In contrast, another meta-analysis showed that WBV significantly reduced lower limb spasticity [16]. A possible explanation for this inconsistency may be due to the heterogeneity of inclusion criteria regarding stroke characteristics, such as the course of disease. In the meta-analysis by Zhang et al. [16], most of the participants included in the subgroup analysis of lower limb spasticity were patients with acute and subacute stroke (0–6 months), and most of these included studies were reported in a non-English language. In addition, insufficient information about hip spasticity hindered our subgroup analysis, which might be due to the difficulty of measuring MAS in this complex joint structure, such as the hip adductors and internal rotators [11]. Therefore, further study is needed to determine the efficacy of VT on lower limb spasticity, especially in the hip, and gait performance in patients with PSS.

Actually, the distribution of spasticity on the hemiplegic side may vary among individual stroke patients. In addition to limb spasticity, trunk muscle spasticity is also common in stroke patients and are characterized by different abnormal patterns in the thoracolumbar region, such as tension or flexion [45]. Li et al. [45] reported that WBV can significantly improve the trunk muscle spasticity in people with stroke. Currently, there are very limited reports on VT for trunk muscle spasticity in people with stroke. Considering the lack of uniform evaluation criteria for trunk muscle spasticity, it is difficult to draw conclusions about the efficacy of VT on trunk spasticity in stroke patients at present.

Several studies have shown that the presence of spasticity is not influenced by gender, age, or affected hemisphere [46–48]. However, the relationship between other participant characteristics and the occurrence of spasticity, as well as their impact on the efficacy of VT for spasticity, remains unclear. Our results revealed that although the effect size was larger in the European group compared to the Asian group, the differences between these groups were not statistically significant. It is important to acknowledge certain limitations, including insufficient sample size, variations in study design, and subgroup analyses based on population categorization that did not effectively reduce heterogeneity. Therefore, further investigation is warranted to ascertain the influence of participant characteristics, such as population classification, on the efficacy of VT for individuals with PSS.

Regarding the long-term effect of VT, our subgroup analysis by time of assessment suggested that the lasting efficacy of VT on spasticity might be limited. In previous studies, Noma et al. [39] found that the inhibitory effect of LMV on upper limb spasticity in stroke patients lasted for 30 min after treatment. Similarly, Marconi et al. [23] found that the anti-spasticity effect of LMV on the upper limbs of stroke patients lasted for 2 weeks after treatment. Clinical experience suggests that spasticity symptoms are more likely to return to their pre-treatment state under the stimulation of various subjective and objective factors, such as sneezing, emotional excitement, excessive exertion, cold, pain stimulation. Therefore, determining the duration of the sustained effect of VT is crucial for clinical practice decisions, and more high-quality RCTs are needed to enrich the evidence on the time-response relationship.

In addition, post-stroke pain and PSS are often considered correlated predictors, with an increase in pain associated with spasticity and vice versa [49]. Our research showed that VT significantly reduced post-stroke pain. This may be due to changes in the activity of body receptors, such as annular bodies and Ruffinian bodies that affect skin sensory pain due to vibration stimulation, thereby increasing the sensory threshold of skin pain [36, 37]. Suitable vibration stimuli applied to muscles or tendons can produce analgesic effects at the time of application and immediately after cessation.

### Limitations

This study had several limitations. Firstly, many clinical studies do not classify participants by stroke type, affected brain region, and severity of spasticity, resulting in considerable heterogeneity between individuals. Given that stroke is a heterogeneous disease, its prognosis often depends on the location and size of the penumbra. Therefore, more research is needed to confirm the effectiveness of VT in certain types of patients. Secondly, although the spasticity assessment method based on electromyography and biomechanical analysis has been proven to have good reliability and validity, the number of randomized controlled trials (RCTs) using these assessment methods to explore VT for PSS is limited, and we acknowledge that the lack of analysis of such outcome measures is one of the limitations of our study. Finally, due to data unavailability and exclusion of non-English publications, our sample size was limited, which may introduce some selection bias. The overall effects and conclusions derived from subgroup analyses would be more statistically convincing with more VT related trials, and we strongly recommend that future trials address this issue.

## Conclusion

Our study demonstrates that VT can effectively alleviate spasticity and pain, and improve motor function in people with PSS, particularly in the upper limbs. Evidence for the effectiveness of VT in the lower limbs, including spasticity and gait performance, is relatively limited. Subgroup analysis suggests that the optimal parameters of VT for anti-spasticity treatment, such as vibration type, frequency, duration, sessions, and control method, require further investigation through more RCTs. Furthermore, our findings indicate a short-term anti-spasticity effect following VT, but evidence of a long-term benefit is lacking. Therefore, in clinical practice, effective VT should take into account the actual functional status of the individual, appropriate treatment parameters, and potential adverse reactions to improve treatment safety.

### Methods

This study was conducted in accordance with the guidelines of the Cochrane Selection Manual and the Preferred Reporting Items for Systematic Reviews and Meta-Analysis (PRISMA) [50, 51]. The protocol was registered in the International Prospective Register of Systematic Reviews with registration number CRD42023397031.

### Search strategy

Searches were conducted in PubMed, Embase, Cochrane Library, Physiotherapy Evidence Database, and Web of Science from the inception to October 2022. The database search was performed following the PICOS (participant, intervention, control, outcome, study design) principles and used the AND operator to combine the search results for PSS, VT, and RCTs. The complete search strategies for PubMed, Embase, and Cochrane Library are presented in Additional file 8: Table S1. We also searched the reference lists of previously published studies. Two evaluators conducted this process independently, and in case of discrepancies, a group discussion with multiple reviewers was held until a consensus was reached.

### Eligibility criteria

First, title and abstract screening was conducted, and articles that did not meet the inclusion criteria were excluded. If the relevance of a literature was unclear, a full-text evaluation was performed. Two reviewers conducted this process independently based on the eligibility criteria. The inclusion criteria were: (1) participants: individuals diagnosed with stroke and experiencing spasticity; (2) interventions: VT (WBV or LMV); (3) comparisons: routine rehabilitation, sham VT, or no VT as control measures; (4) primary outcome: spasticity measured by the modified Ashworth scale (MAS); the article must have reported raw data (or graphs from which data can be extracted) related to MAS; (5) secondary outcomes: pain, motor function, gait, and adverse effects; pain was assessed using the visual analog scale or verbal numerical rating scale; motor function was assessed using the Fugl-Meyer assessment, motricity index, or Wolf motor function test; gait was evaluated by the 6-minute walk test, 10-meter walk test or timed up and go test; (6) study design: RCTs published in English. The exclusion criteria were: (1) abstracts or conference papers; (2) articles published without peer review.

### Data extraction

Two independent reviewers used the same Excel spreadsheet to perform the data extraction process. If there were any discrepancies in the extracted data, group discussions were held to ensure the accuracy of the data. The extracted data included: (1) study characteristics (such as country, sample size); (2) participants characteristics (including gender, age, side of lesion, stroke type, time since stroke); (3) intervention characteristics (including vibration type, frequency, duration, sessions, spasticity location, and control therapy); (4) outcome data, including primary and secondary outcomes measured by related clinical scales for each treatment group. The measurement time point, follow-up time, and adverse events were also recorded. In the case of multi-arm trials with multiple treatment groups (such as different frequencies), each treatment group was considered a separate trial.

### Assessment of risk of bias

The Cochrane Collaboration tool was utilized to assess the risk of bias for included studies [50, 52]. This tool comprises 7 aspects, including random selection methods, allocation concealment, blinding of researchers and subjects, blinding of research outcomes, integrity of outcome data, selective reporting of research results, and other sources of bias. Following the bias risk assessment criteria, two independent evaluators classified each aspect as "low risk bias," "high risk bias," or "unclear."

### Data analysis

Meta-analysis was performed using Stata SE 17.0 (StataCorp, College Station, TX, USA). The effect of VT on PSS was demonstrated by calculating the standardized mean difference (SMD) and the corresponding 95% confidence interval (CI). The random-effects model with DerSimonian-Laird was used to measure the pooled weighted effect size, and statistical significance was defined as *P* < 0.05. Heterogeneity was analyzed using the I square (*I*^2^) index and the Cochran *Q* test. An *I*^2^ value greater than 50% and *P*-value less than 0.1 indicated significant heterogeneity among the included studies. Funnel plots and the Egger test were applied to estimate potential publication bias. An asymmetric funnel plot or a *P*-value less than 0.05 indicated significant publication bias. Sensitivity analysis with leave-one-out was conducted to evaluate the robustness of the results.

Subgroup analyses were performed to identify the sources of heterogeneity and explore the factors that influence the effect of VT on spasticity in PSS: (1) vibration subtype (WBV vs. LMV); (2) vibration frequency (≤ 20 Hz vs. > 20 Hz); (3) duration of each session (5 min vs. 5–30 min vs. 30 min); (4) number of sessions (single-session vs. multi-session); (5) frequency of sessions (2 days/week vs. 3 days/week vs. 5 days/week); (6) control therapy (sham VT vs. none VT); (7) distribution of spasticity (shoulder vs. elbow vs. wrist vs. knee vs. ankle); (8) population classification (European vs. Asian).

### Supplementary Information

**Additional file 1: Figure S1.** Forest plot illustrating the effect size of the multi-session VT on spasticity.

**Additional file 2: Figure S2.** Forest plots illustrating the long-term effects of VT on spasticity.

**Additional file 3: Figure S3.** Forest plots illustrating the effects of VT on pain.

**Additional file 4: Figure S4.** Forest plots illustrating the effects of VT on motor function.

**Additional file 5: Figure S5.** Forest plots illustrating the effects of VT on gait.

**Additional file 6: Figure S6.** Publication bias for the effect of VT on spasticity.

**Additional file 7: Figure S7.** Sensitivity analysis for the effect of VT on spasticity.

**Additional file 8: Table S1.** The full strategies for PubMed, Cochrane Library, and Embase.

## Data Availability

The datasets supporting the conclusions of this article are included within the article.

## Abbreviations

10MWT: 10-meter walk test
CI: Confidence interval
FMA: Fugl-Meyer assessment
LE: Lower limbs
LMV: Local muscle vibration
MAS: Modified Ashworth scale
MI: Motricity index
PICOS: Participant, intervention, comparison, outcome, and study design
PSS: Post-stroke spasticity
RCTs: Randomized controlled trials
SMD: Standardized mean difference
TUGT: Timed up and go test
UE: Upper limbs
VAS: Visual analog scale
VNRS: Verbal numerical rating scale
VT: Vibration therapy
WBV: Whole-body vibration
WMFT: Wolf motor function test
