# Supplementary figures and images for: Effects of vibration therapy for post-stroke spasticity: a systematic review and meta-analysis of randomized controlled trials
Source: Biomed Eng Online. 2023 Dec 12;22:121. doi: 10.1186/s12938-023-01176-x (PMC10714496; doi:10.1186/s12938-023-01176-x)

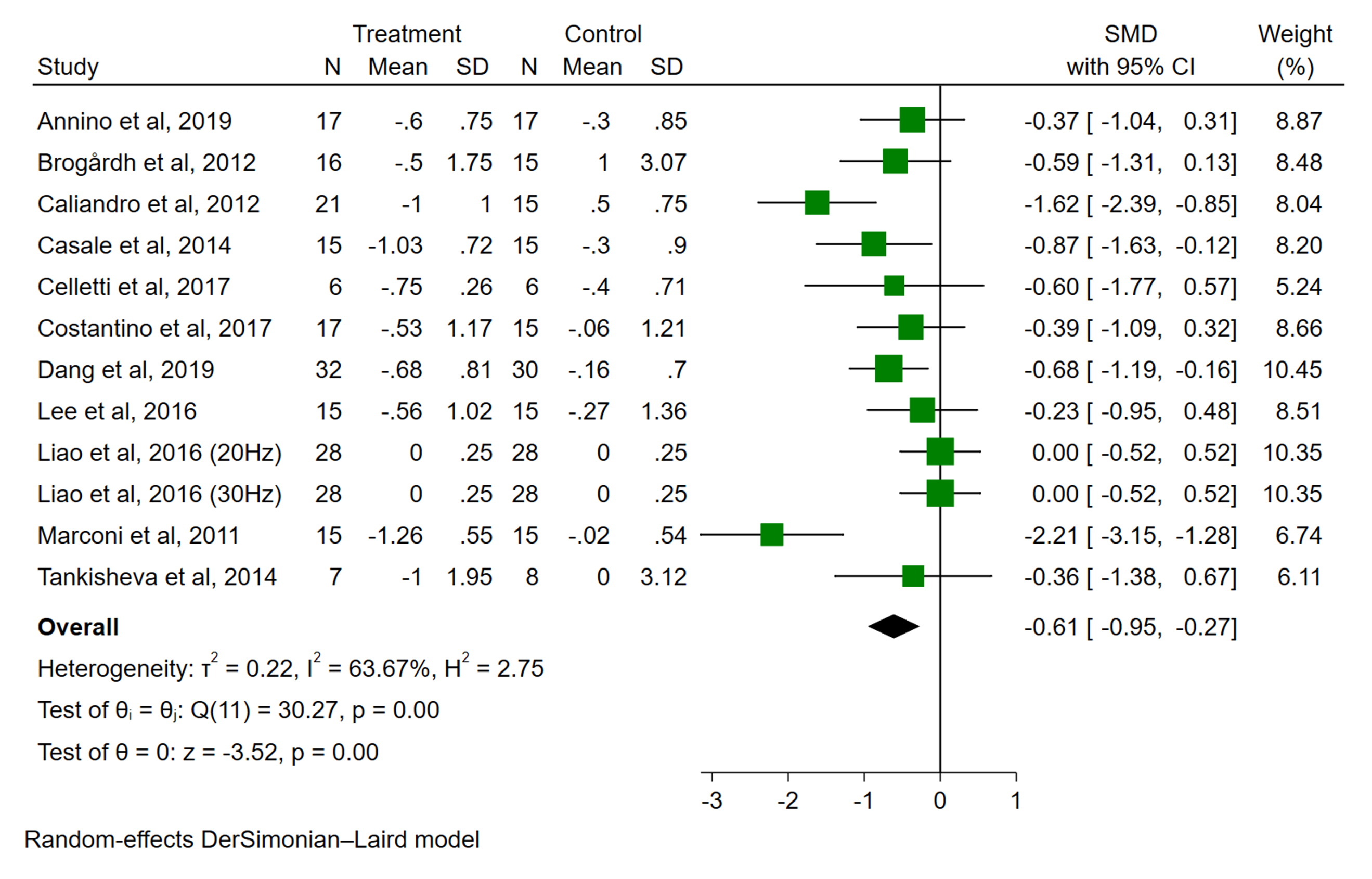

Supplement: Supplementary file 1 — Additional file 1: Figure S1. Forest plot illustrating the effect size of the multi-session VT on spasticity. [file 12938_2023_1176_MOESM1_ESM.tif]

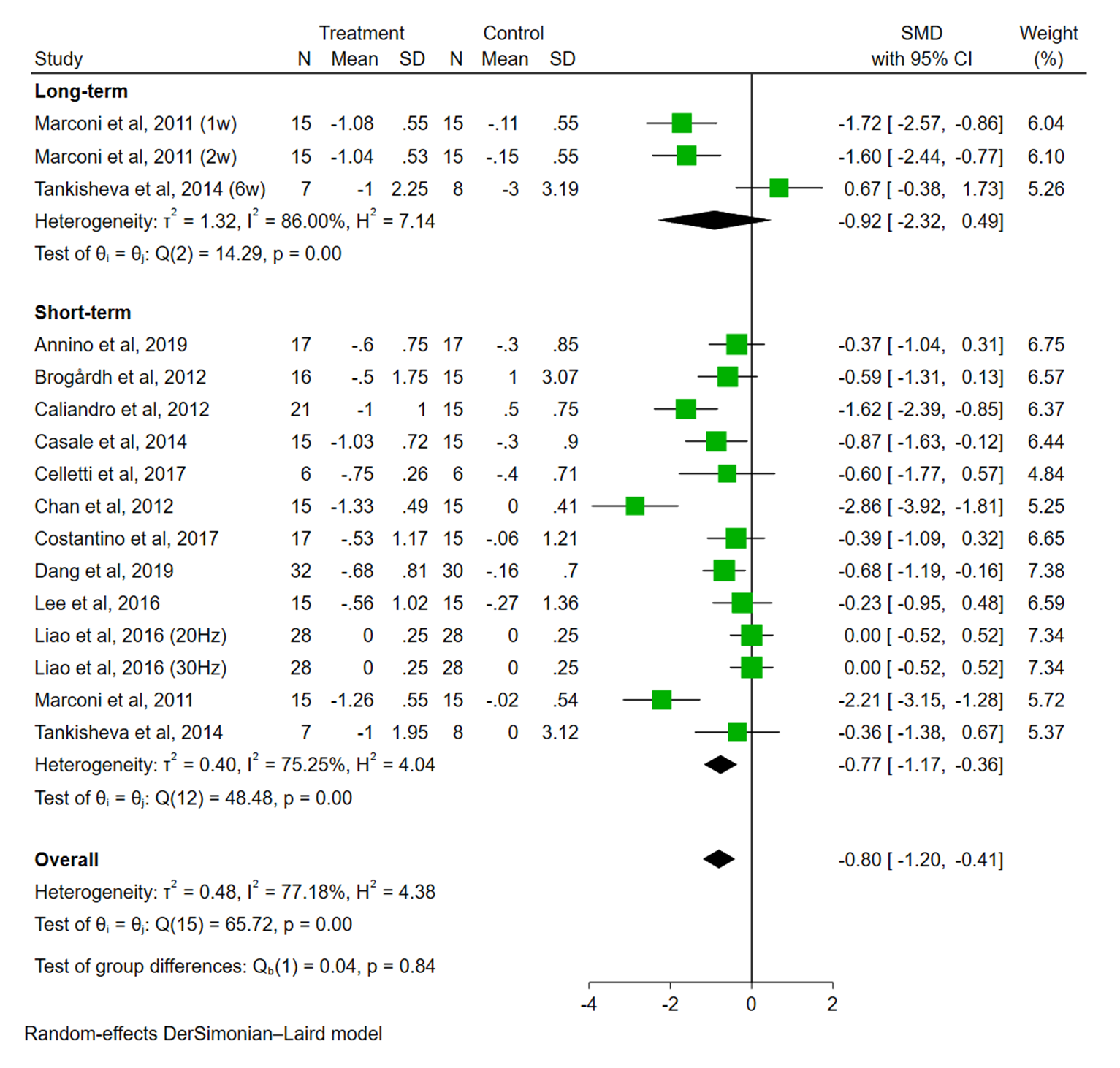

Supplement: Supplementary file 2 — Additional file 2: Figure S2. Forest plots illustrating the long-term effects of VT on spasticity. [file 12938_2023_1176_MOESM2_ESM.tif]

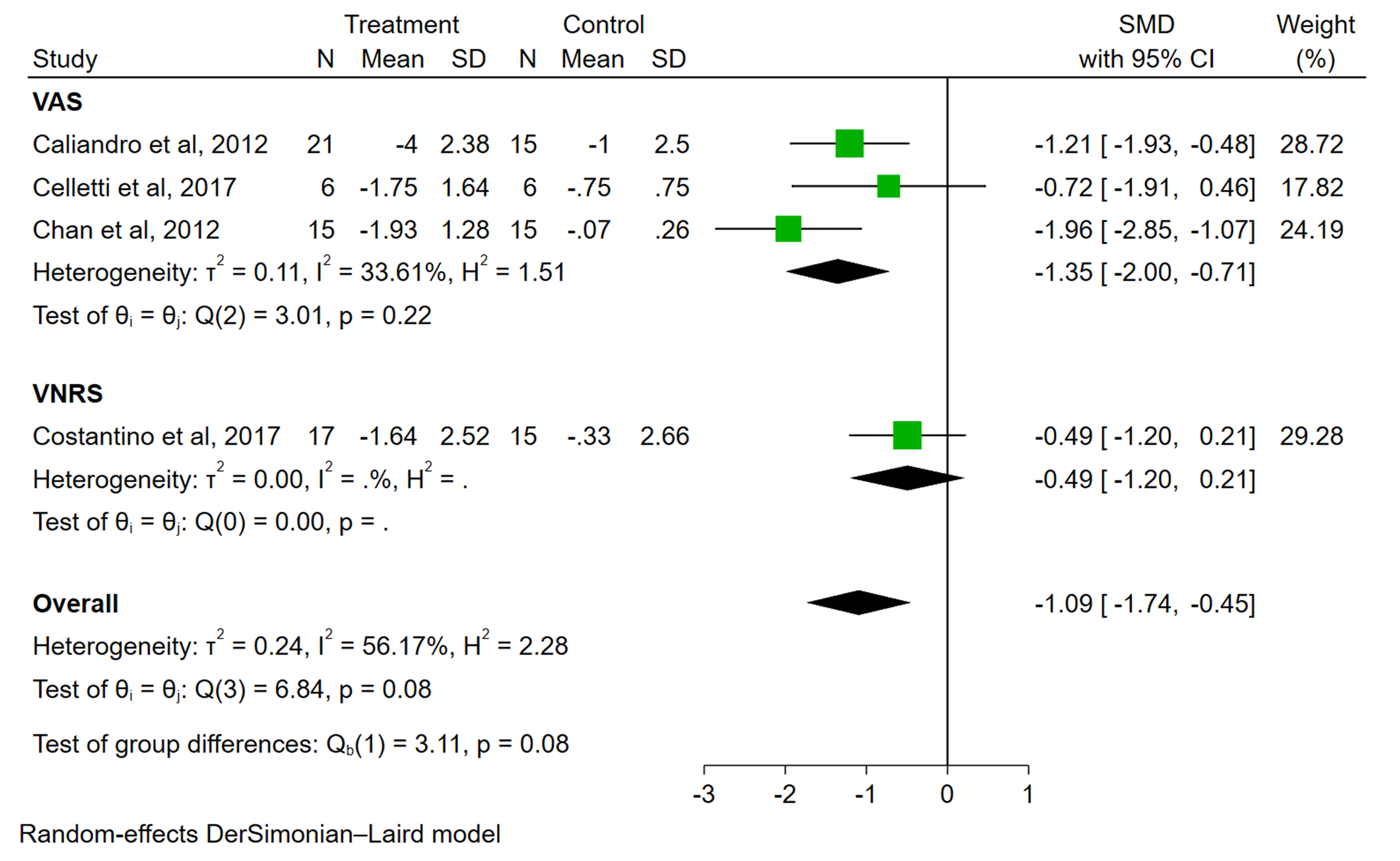

Supplement: Supplementary file 3 — Additional file 3: Figure S3. Forest plots illustrating the effects of VT on pain. [file 12938_2023_1176_MOESM3_ESM.tif]

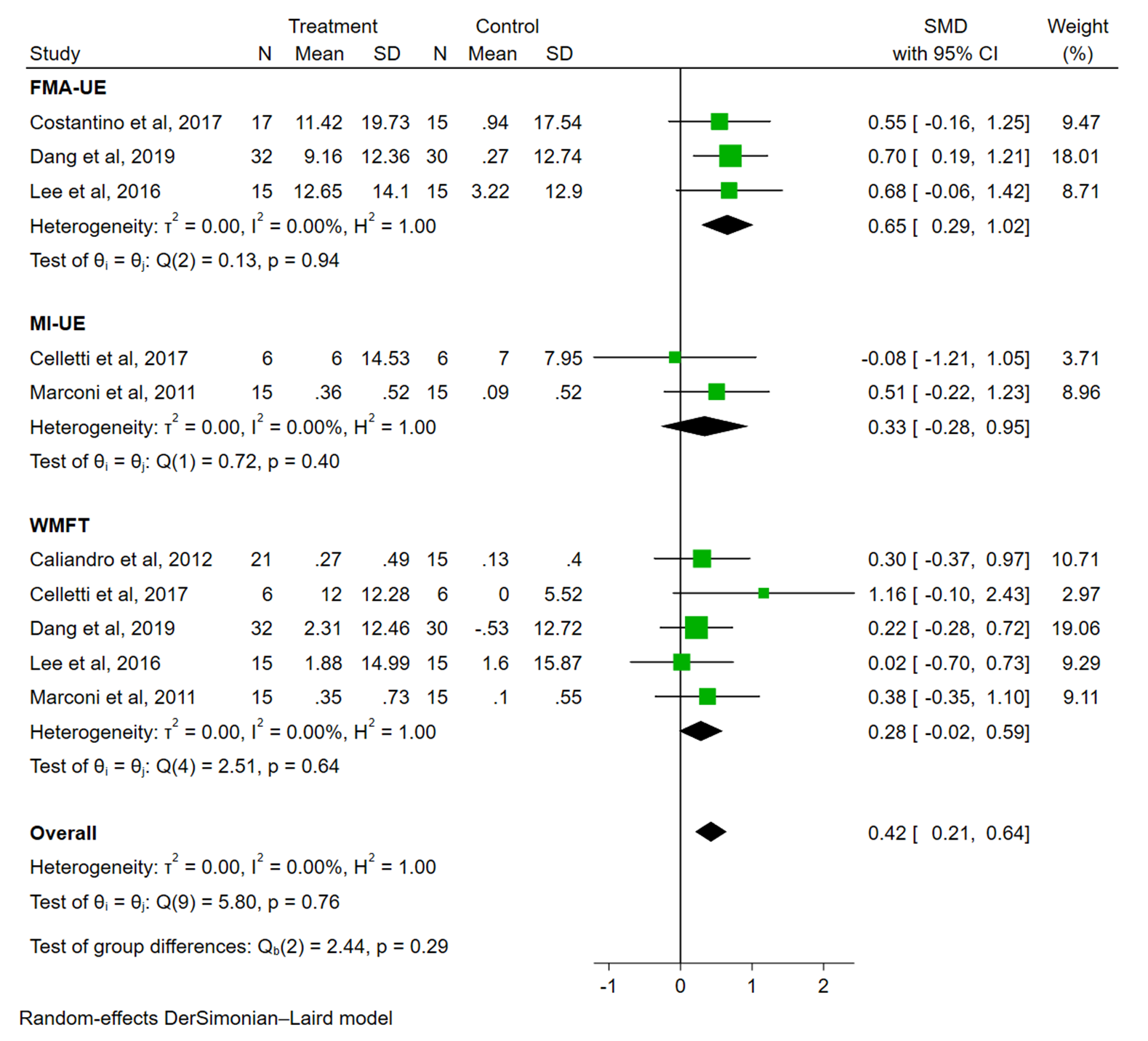

Supplement: Supplementary file 4 — Additional file 4: Figure S4. Forest plots illustrating the effects of VT on motor function. [file 12938_2023_1176_MOESM4_ESM.tif]

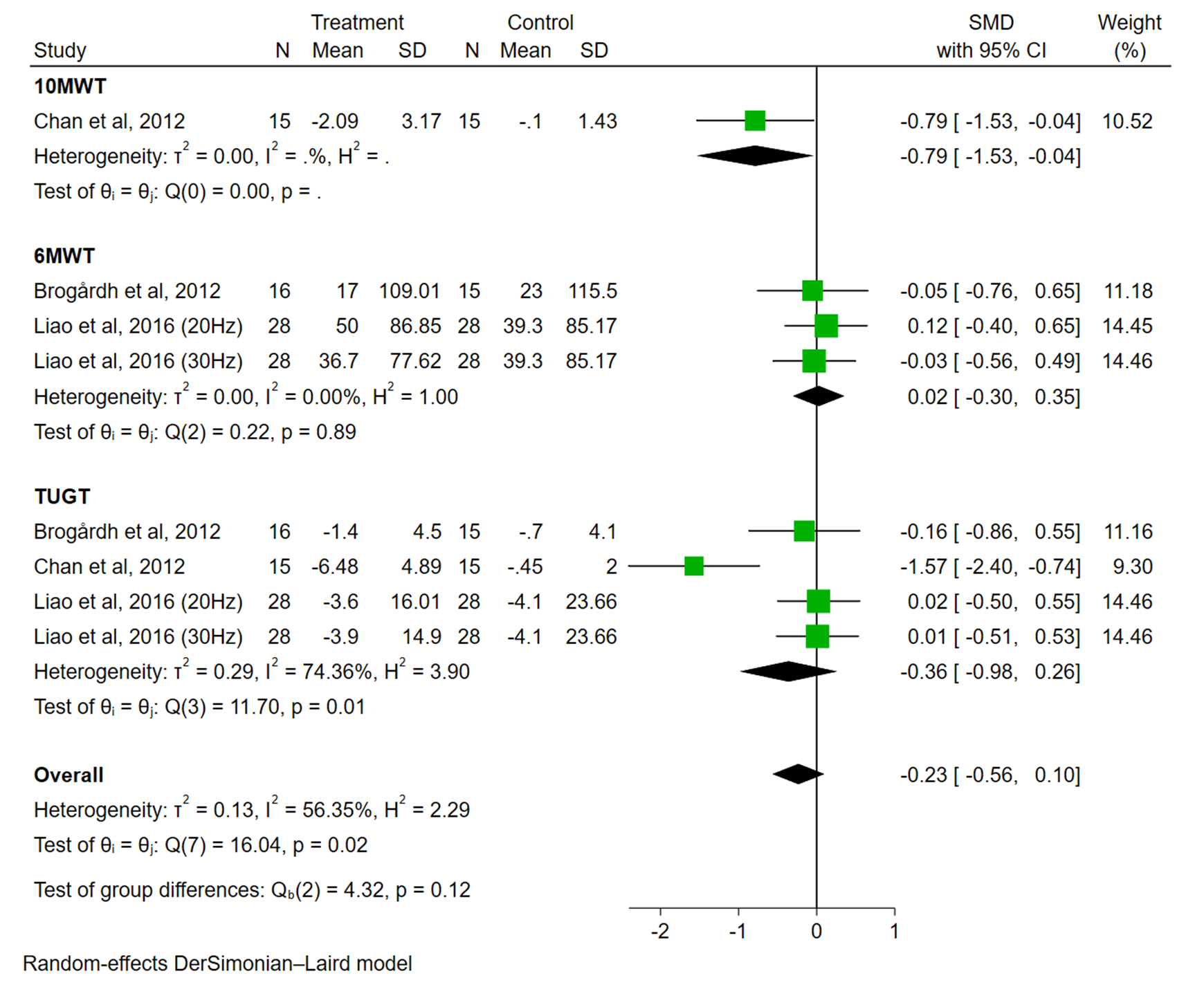

Supplement: Supplementary file 5 — Additional file 5: Figure S5. Forest plots illustrating the effects of VT on gait. [file 12938_2023_1176_MOESM5_ESM.tif]

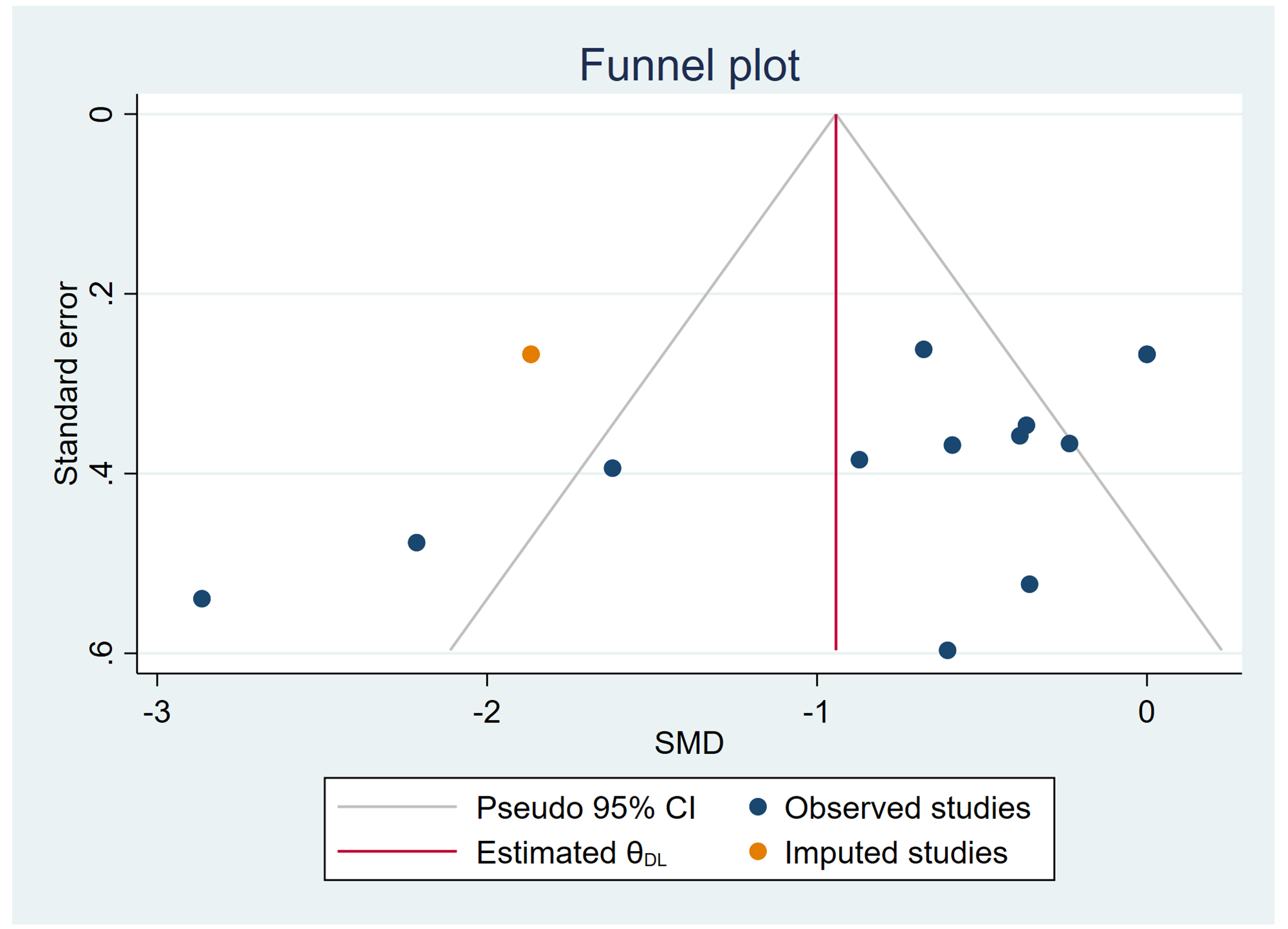

Supplement: Supplementary file 6 — Additional file 6: Figure S6. Publication bias for the effect of VT on spasticity. [file 12938_2023_1176_MOESM6_ESM.tif]

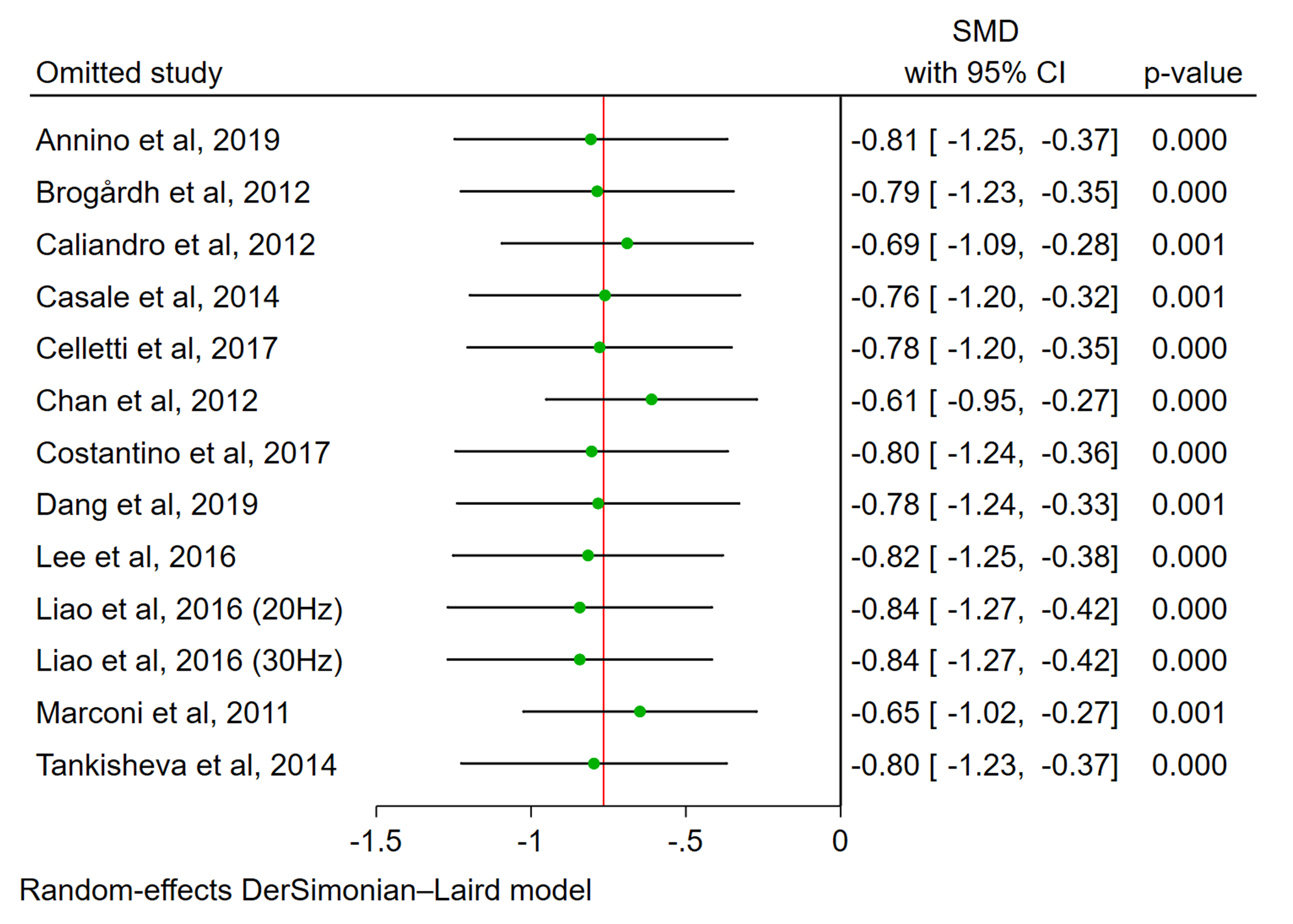

Supplement: Supplementary file 7 — Additional file 7: Figure S7. Sensitivity analysis for the effect of VT on spasticity. [file 12938_2023_1176_MOESM7_ESM.tif]
