# Supplementary material for: Effects of vibration therapy for post-stroke spasticity: a systematic review and meta-analysis of randomized controlled trials
Source: Biomed Eng Online. 2023 Dec 12;22:121. doi: 10.1186/s12938-023-01176-x (PMC10714496; doi:10.1186/s12938-023-01176-x)
Supplement: Supplementary file 8 — Additional file 8: Table S1. The full strategies for PubMed, Cochrane Library, and Embase. [file 12938_2023_1176_MOESM8_ESM.docx]

**Additional file 8: Table S1.** The full strategies for PubMed, Cochrane Library, and Embase

| **Database** | **Search number** | **Search strategy** |
| --- | --- | --- |
| PubMed | #1 | Cerebrovascular disorders[Mesh] |
|  | #2 | Brain injuries[Mesh] OR Brain Damage, Chronic[Mesh] |
|  | #3 | Hemiplegia[Mesh] OR Paresis[Mesh] |
|  | #4 | Gait Disorders, Neurologic[Mesh] |
|  | #5 | stroke*[Title/Abstract] OR cva[Title/Abstract] OR poststroke[Title/Abstract] OR post‐stroke[Title/Abstract] |
|  | #6 | cerebrovasc*[Title/Abstract] OR cerebral vascular[Title/Abstract] |
|  | #7 | cerebral[Title/Abstract] OR cerebellar[Title/Abstract] OR brain*[Title/Abstract] OR vertebrobasilar[Title/Abstract] |
|  | #8 | infarct*[Title/Abstract] OR ischaemi*[Title/Abstract] OR ischemi*[Title/Abstract] OR thrombo*[Title/Abstract] OR emboli*[Title/Abstract] OR apoplexy[Title/Abstract] |
|  | #9 | #7 AND #8 |
|  | #10 | cerebral[Title/Abstract] OR brain[Title/Abstract] OR subarachnoid[Title/Abstract] |
|  | #11 | haemorrhage[Title/Abstract] OR hemorrhage[Title/Abstract] OR haematoma[Title/Abstract] OR hematoma[Title/Abstract] OR bleed*[Title/Abstract] |
|  | #12 | #10 AND #11 |
|  | #13 | hemipleg*[Title/Abstract] OR paresis[Title/Abstract] OR paretic[Title/Abstract] OR brain injur*[Title/Abstract] |
|  | #14 | #1 OR #2 OR #3 OR #4 OR #5 OR #6 OR #9 OR #12 OR #13 |
|  | #15 | Vibration[Mesh] |
|  | #16 | Vibration*[Title/Abstract] |
|  | #17 | #15 OR #16 |
|  | #18 | (randomized controlled trial[pt] OR controlled clinical trial[pt] OR randomized[tiab] OR placebo[tiab] OR clinical trials as topic[mesh:noexp] OR randomly[tiab] OR trial[ti]) NOT (animals [mh] NOT (humans [mh] AND animals[mh])) |
|  | #19 | #14 AND #17 AND #18 |
| Cochrane Library | #1 | MeSH descriptor: [Cerebrovascular Disorders] explode all trees |
|  | #2 | MeSH descriptor: [Brain Injuries] explode all trees |
|  | #3 | MeSH descriptor: [Brain Damage, Chronic] explode all trees |
|  | #4 | MeSH descriptor: [Hemiplegia] explode all trees |
|  | #5 | MeSH descriptor: [Paresis] explode all trees |
|  | #6 | MeSH descriptor: [Gait Disorders, Neurologic] explode all trees |
|  | #7 | (stroke* or cva or poststroke or post‐stroke):ti,ab,kw OR (cerebrovasc* or cerebral vascular):ti,ab,kw |
|  | #8 | (Cerebral or cerebellar or brain* or vertebrobasilar):ti,ab,kw AND (infarct* or ischaemi* or ischemi* or thrombo* or emboli* or apoplexy):ti,ab,kw |
|  | #9 | (cerebral or brain or subarachnoid):ti,ab,kw AND (haemorrhage or hemorrhage or haematoma or hematoma or bleed*):ti,ab,kw |
|  | #10 | (hemipleg* or paresis or paretic or brain injur*):ti,ab,kw |
|  | #11 | #1 OR #2 OR #3 OR #4 OR #5 OR #6 OR #7 OR #8 OR #9 OR #10 |
|  | #12 | MeSH descriptor: [Vibration] explode all trees |
|  | #13 | (vibration*):ti,ab,kw |
|  | #14 | #12 OR #13 |
|  | #15 | #11 AND #14 |
| Embase | #1 | 'cerebrovascular disease'/exp |
|  | #2 | 'brain injury'/exp |
|  | #3 | 'chronic brain disease'/exp |
|  | #4 | hemiplegia'/exp |
|  | #5 | 'paresis'/exp |
|  | #6 | 'neurologic gait disorder'/exp |
|  | #7 | stroke*:ab,ti OR cva:ab,ti OR poststroke:ab,ti OR post‐stroke:ab,ti OR cerebrovasc*:ab,ti OR 'cerebral vascular':ab,ti |
|  | #8 | (cerebral:ab,ti OR cerebellar:ab,ti OR brain*:ab,ti OR vertebrobasilar:ab,ti) AND (infarct*:ab,ti OR ischaemi*:ab,ti OR ischemi*:ab,ti OR thrombo*:ab,ti OR emboli*:ab,ti OR apoplexy:ab,ti) |
|  | #9 | (cerebral:ab,ti OR brain:ab,ti OR subarachnoid:ab,ti) AND (haemorrhage:ab,ti OR hemorrhage:ab,ti OR haematoma:ab,ti OR hematoma:ab,ti OR bleed*:ab,ti) |
|  | #10 | hemipleg*:ab,ti OR paresis:ab,ti OR paretic:ab,ti OR 'brain injur*':ab,ti |
|  | #11 | #1 OR #2 OR #3 OR #4 OR #5 OR #6 OR #7 OR #8 OR #9 OR #10 |
|  | #12 | 'vibration'/exp |
|  | #13 | vibration*:ab,ti |
|  | #14 | #12 OR #13 |
|  | #15 | 'crossover procedure':de OR 'double-blind procedure':de OR 'randomized controlled trial':de OR 'single-blind procedure':de OR random*:de,ab,ti OR factorial*:de,ab,ti OR crossover*:de,ab,ti OR ((cross NEXT/1 over*):de,ab,ti) OR placebo*:de,ab,ti OR ((doubl* NEAR/1 blind*):de,ab,ti) OR ((singl* NEAR/1 blind*):de,ab,ti) OR assign*:de,ab,ti OR allocat*:de,ab,ti OR volunteer*:de,ab,ti |
|  | #16 | #11 AND #14 AND #15 |
